# Effects of Compression Tights on Recovery Parameters after Exercise Induced Muscle Damage: A Randomized Controlled Crossover Study

**DOI:** 10.1155/2019/5698460

**Published:** 2019-01-08

**Authors:** Michael Hettchen, Katharina Glöckler, Simon von Stengel, Andrea Piechele, Helmut Lötzerich, Matthias Kohl, Wolfgang Kemmler

**Affiliations:** ^1^Institute of Medical Physics, Friedrich-Alexander University of Erlangen-Nürnberg (FAU), Erlangen, Germany; ^2^Institute of Outdoor Sports and Environmental Science, German Sports University Cologne, Cologne, Germany; ^3^Department of Medical and Life Sciences University of Furtwangen, Schwenningen, Germany

## Abstract

**Introduction:**

Recent meta-analyses on compression garments have reported faster recovery of muscle function particularly after intense eccentric power or resistance exercise. However, due to the complex interaction between cohorts included, exercises involved and compression applied, recovery length and modalities, and outcome parameters selected, only limited practical recommendations can be drawn from these studies. Thus, our aim was to determine the effect of compression tights on recovery from high mechanical and metabolic stress monitored over a longer recovery period.

**Material and Methods:**

Using a crossover design, 19 resistance-trained 4^th^/5^th^ Division German handball players (31.3±7.7 years; 24.1±3.8 kg/m^2^) were randomly assigned at the start of the project to the compression tight (recovery-pro-tights, cep, Bayreuth, Germany) or the control group. Immediately after a combined lower extremity resistance training and electromyostimulation, participants had to wear compression tights. Compression was applied initially for 24 h and then 12 h intermitted by 12 h of nonuse for a total of 96 h. Primary study endpoint was maximum isokinetic hip/leg-extensor strength (MIES) as determined by a leg-press. Secondary endpoint was lower extremity power as assessed by a counter movement jump. Follow-up assessments were conducted 24, 48, 72, and 96 h postexercise. Outcomes were analyzed using a linear mixed effect model with spherical symmetric within-condition correlation.

**Results:**

All 19 participants underwent their allocated treatment and passed through the project strictly according to the study protocol. MIES demonstrated significantly (p=0.003) lower overall reductions (155 N) after wearing compression tights. In parallel, lower extremity power significantly (p<0.001) varies between both conditions with lower reductions in favor of the compression condition. Of importance, full recovery for lower extremity muscle strength or power was still not reached 96 h postexercise.

**Conclusion:**

Based on our results we recommend athletes wear compression tights for faster recovery, particularly after intense exercise with a pronounced eccentric aspect.

## 1. Introduction

In the last fifteen years, the wearing of compression garments in various amateur and professional sports has become ever more frequent. However, although a limited number of studies indicate some evidence that physical performance might be increased by compression garments (review in [1, 2]), current focus of research is increasingly on recovery after intense muscular performance (review in [3–5]). In summary, recent reviews and meta-analyses reported faster recovery of muscle function [3] after exercise particularly muscle damage induced by power or resistance exercise (exercise-induced muscle damage: EIMD) [5, 6]. However, considering the heterogeneity of the few studies in this area, we are not convinced whether meta-analysis might be able to conclude the effect of compression garment on recovery from exercise-induced muscle soreness or damage. Indeed, reviewing the underlying trials there are some methodical flaws that might seriously confound and/or impede proper interpretation of the results. The most relevant bias might be that many studies applied exercise protocols unable or at least suboptimal to generate EIMD or even profound muscular soreness (overview in [3]). A good example is to apply a running intervention for runners. However, even when conducted with higher intensity, the type and composition of this “damaging protocol” will be too close to the usual exercise strain to generate relevant EIMD. Correspondingly, compression-induced effects on only slightly affected parameters might be negligible. Further, study outcomes often focus on parameters (e.g., lactate, creatine kinase, [7, 8]) at least suboptimum for validating recovery. Additionally, the minority of trials (<20-25%; e.g., [9–14]) monitor recovery periods >48 h. Thus, the aim of the study was to determine the effect of compression tights on relevant parameters of recovery applying a conscientious methodological and biometrical approach. In summary, we hypothesize that performance parameters, perceived physical state, and blood parameters related to EIMD were positively affected during recovery from a single bout of strenuous resistance-type exercise by wearing compression tights. Our primary hypothesis was that compression tights significantly increase recovery of performance parameters after EIMD-generating exercise compared with control, as validated by maximum isokinetic hip- and leg-extensor strength.

## 2. Materials and Methods

The study was designed and realized by the Institute of Medical Physics, Friedrich-Alexander University of Erlangen-Nürnberg (FAU), Germany, in close cooperation with the German Sports University Cologne, (DSHS), Cologne, Germany. All parts of the project were conducted between December 2017 and February 2018 and complied with the Helsinki Declaration “Ethical Principles for Medical Research Involving Human Subjects” [15]. The ethics committee of the German Sports University Cologne, Germany, approved this study (registration number: 024/2016). After detailed information, all participants gave their written informed consent. The study was fully registered under clinicaltrials.gov NCT03417323. In this article, we follow the Consolidated Standards of Reporting Trials (CONSORT) guideline for reporting parallel group randomized trials [16].

### 2.1. Study Design

**Figure 1:**
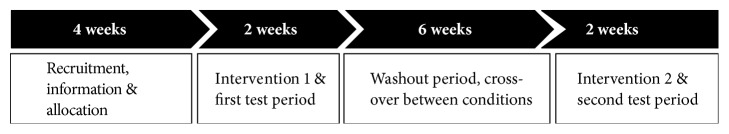
Study design.

Using a crossover design, participants were randomly assigned to the compression garment condition that started with the treatment condition during phase 1 or to the control condition that started without compression garment after the intervention (i.e., the single session of exercise applied in this study). In parallel, participants who started with the control condition during phase 1 then switched to the treatment condition in phase 2 and vice versa. Correspondingly, the study was structured into two interventions, one in December 2017 and one in January 2018 separated by a 6-7 week wash-out period (Figure 1).

### 2.2. Participants

Using personal contacts to handball teams playing in the 4^th^ and 5^th^ German Divisions, we gave detailed study information including the most relevant eligibility criteria (e.g., training, health status) during two information meetings. Twenty-eight healthy male adults 20-50 years old, living in the area of Erlangen-Nürnberg, Germany, were interested and were further assessed for eligibility. Applying the inclusion criteria: (a) more than 5 years of experience in competition ball sports with corresponding discipline-specific resistance exercise once a week and exclusion criteria: (a) diseases and medication affecting muscle metabolism; (b) contraindication for whole-body electromyostimulation (WB-EMS) application (e.g., cardiac pacemaker) or heavy resistance exercise (e.g., knee/hip arthrosis); (c) application of WB-EMS during the last 6 months; (d) absence during the testing periods and (e) the intention to apply types of exercise known to induce severe muscle pain (i.e., marathon run) during the study period, finally 19 participants were included in the study.

### 2.3. Intervention

Due to the very intense muscular strain induced by the exercise protocol [17] the exercise tests and the recovery period were closely supervised by medical staff.

#### 2.3.1. Exercise Intervention

The exercise interventions were conducted between 8:00 and 10:00 on Sunday or Monday morning at the Institute of Medical Physics, FAU, under close medical supervision. In both periods (i.e., December and January), the identical high intensity resistance exercise (RT) with special emphasis on the eccentric part of the movement was applied. Exercises (Figure 3) focus on lower extremities, further WB-EMS application for the gluteus, thigh, and calf muscles superimpose the voluntary load on the corresponding regions. In detail, using a circuit mode, two sets of 8-10 repetitions of lunges (both sides immediately in succession), unilateral calf raises (both sides immediately in succession), and squats were prescribed. Using dumbbells, participants were required to increase the load to realize the prescribed work to momentary failure per exercise [18] in the range of 8-10 reps. We scheduled a time under tension with 4 sec eccentric—1 s isometric—and an explosive concentric phase. We set a break of 2 s between the reps in order to synchronize exercises with the impulse phase of the EMS. Recovery between exercises averages 60 s; time between the two rounds of the circuit averaged 2 min. Participants were carefully instructed on how to conduct the exercises before the combined RT/WB-EMS intervention.

The EMS equipment (miha bodytec GmbH, Gerstenhofen, Germany) used in this project consisted of various vest, hip, leg, and arm electrodes that allowed a simultaneous stimulation of all the main muscle groups with an stimulation area of up to 2,800 cm^2^ with regionally dedicated specification. However, as reported in this project we focus on the gluteus, thigh, and calf regions (Figure 3), i.e., the scope of application of the compression tights. Using our standard WB-EMS protocol [19], impulse frequency was set at 85 Hz with an impulse width of 350 *μ*s. We applied an intermitted rectangular impulse pattern with 6 s impulse and 2 s impulse break. During the impulse phase the single repetitions of the voluntary exercises were performed. (Impulse) intensity was individually adapted for each lower body region in close interaction between the participant and the instructor. We aimed to achieve an impulse intensity that just allowed proper conducting of the voluntary movements under the premise of work to momentary failure. In order to quantify this specification we asked for the participants' perceived exertion rating immediately after the exercise tests using the Borg CR 10 scale of perceived exertion (RPE) [20].

Participants who underwent the control condition after the exercise intervention were asked to maintain their habitual lifestyle without any attempt to reduce muscle pain or discomfort by recognized methods.

Further, all the participants were instructed not to exercise 72 h prior to the exercise intervention or during the 96 h testing phase.

#### 2.3.2. Recovery Intervention

As reported, participants were randomly assigned to start with the treatment (i.e., compression tights) or control condition. Participants of the treatment condition started to wear the compression tights immediately after the exercise intervention for 24 h. After this period, ≈12 h of wearing compression tights (8^00^-10^00^ to 20^00^-22^00^) were intermitted by ≈12 h phases without compression tights (20^00^-22^00^ to 8^00^-10^00^). Of importance, compression garments were not worn during the 24, 48, 72, and 96 h follow-up tests.

#### 2.3.3. Compression Garments

We used compression tights (recovery pro tights, cep, Bayreuth, Germany) that cover hip, thigh, and calf from the proximal end of the iliac crest to the distal end of the metatarsus. Tights consisted of 75% polyamide and 25% spandex.

In order to realize the prescribed compression, participants were invited prior to the intervention to determine circumferences at the narrowest point ankle, the widest point of the calf, and the mid-thigh. Using these specifications the corresponding compression tight was selected out of four conventional sizes. However, in eight cases lower leg physiognomy of the participant required customized compression tights which were provided by the manufacturer (cep, Bayreuth, Germany).

Using the HOSYcan System (**Ho**henstein** Sy**stem, Bönnigheim, Germany), compression within the lowest and highest circumference of the suggested size are reported (**Ho**henstein** Sy**stem, Bönnigheim, Germany) to vary between 18.1 and 23.4 mmHg for the most narrow size above the ankle; 19.0-26.2 mmHG for the onset of the calf muscle; 16.3-23.5 mmHg for the highest calf circumference; 9.9-18.1 mmHg two fingerbreadths beneath the fossa poplitea; 7.7-14.3 mmHg at the mid-knee; 9.9-13.9 at mid-thigh and 8.0-12.2 for the region two fingerbreadths beneath the crotch. Due to our failure to determine the compression with completely and properly drawn compression tights at these ankle, calf, and thigh landmarks (see also “European Standards for testing medical hosiery”) using a pneumatic sensor (Kikuhime, MediGroup, Melbourne, Australia), we are unable to validate these data on an individual basis.

### 2.4. Outcome

Changes of maximum isokinetic hip and leg extensor strength as determined by an isokinetic leg-press were considered as the primary study endpoint. Core secondary study endpoint was defined as changes of lower leg power (jumping height) as determined by a counter movement jump on a force plate. Subordinate secondary study endpoints were changes of perceived physical state dimensions as determined by the Perceived Physical State (PEPS) scale, changes of creatine kinase, and myoglobin serum concentration.

#### 2.4.1. Testing

Primary and secondary study outcomes were assessed immediately before (baseline) and 24h, 48h, 72h, and 96 hours after the exercise intervention. We conducted all tests in a blinded fashion; i.e., research assistants were unaware of the status/condition and were not allowed to ask. Baseline and follow-up assessments of the participants were conducted at the same time of day (± 1 h).

Demographic parameters, health risk factors, diseases, medication, lifestyle habits, physical activity, and exercise were sampled by validated baseline questionnaire [21, 22]. In order to determine the self-rated present general condition / perceived physical state of the participants we used the Perceived Physical State (PEPS) questionnaire suggested by Kleinert [23]. Briefly the 20-item version of the PEPS scale was structured into four factors that explained the four dimensions “activation,” “training level,” “health,” and “flexibility” using a scale from 0 (not at all) to 5 (completely). The same questionnaire also asked about acute physical pain sensation and ailments [23].

Height was determined by a stadiometer, (Holtain, Crymych Dyfed., Great Britain); body mass and composition were determined via multisegmental, multifrequency Bio-Impedance Analysis (DSM-BIA, InBody 770, Seoul, Korea) immediately before the first exercise intervention.

Maximum isokinetic strength of the hip/leg extensors and flexors was tested using a ConTrex isokinetic leg-press (Physiomed, Laipersdorf, Germany). Bilateral hip/leg extension and flexion were performed in a sitting, slightly supine position (15°), supported by hip and chest straps. Range of motion was selected between 30° to 90° of the knee angle, with the ankle flexed 90° and positioned on a flexible sliding footplate. The standard default setting of 0.5 m/s was used. After familiarization with the movement pattern, participants were asked to conduct five repetitions with maximum voluntary effort. Participants conducted two trials intermitted by two minutes of rest. We consistently included the highest value for hip/leg extension and hip/leg flexion of the five repetitions and both trials in the data analysis. Applying this approach, reliability for the maximum leg-press test (Test-Retest-Reliability; Intra Class Correlation) was 0.88 (95%-CI: 0.82–0.93) as established in a previous study with 30-50-year-old men [24].

Power of the lower extremities was tested by counter movement jump (CMJ) with hands on the hips (no arm swing) during the test. Starting in an upright position participants were asked to jump as quickly and explosively as possible in order to perform the highest possible jump. We did not restrict countermovement depth; however, we did ask participants to maintain extension in the hip, knee, and ankle joints to prevent any additional flight time by bending their legs. Tests were performed on a force platform (KMP Newton GmbH, Stein, Germany). The software provided by the manufacturer automatically calculates jumping height based on ground reaction forces.

Blood was sampled under nonfasting condition from an antecubital vein before exercise and 24 h, 48 h, 72 h, and 96 h postexercise. In summary, CK and myoglobin were analyzed using the Beckmann Coulter Inc. device and test kits (Brea, Ca, USA). The same research assistant consistently conducted procedures of blood sampling and analysis.

#### 2.4.2. Changes of Trial Outcomes after Trial Commencement

No changes of trial outcomes were conducted after trial commencement.

#### 2.4.3. Sample Size Analysis

Based on our primary study endpoint “maximum dynamic hip-/leg extensor strength,” we considered a difference of percentage changes between compression and noncompression condition (NCC) after 96h of 7.5% (SD 7.5%) as functionally relevant. Applying *α*=0.05 and *β*-1=0.8, in total 16 participants were required to achieve this assumption. However, due to the exhausting test protocol and the postexercise testing in the morning over 4 days that have to be performed twice (with and without compression garments), we anticipated a drop-out rate of about 20%; thus we included all the eligible men (n=19).

#### 2.4.4. Randomization Procedures and Enrollment

Nineteen eligible men were randomly assigned to two conditions that started with or without compression tights during the first test period. Due to the crossover design of the study, we used only a simple but balanced random (1-1) allocation. Supervised by the researcher responsible for the randomization process (SvS), the participants drew lots and allocated themselves to the two conditions. Lots were put in opaque plastic shells (“kinder egg,” Ferrero, Italy) and were drawn by the participants from one bowl in the order of their appearance. Of importance, neither participants nor researchers knew the allocation beforehand. Finally, nine men started with and 10 men started without compression garment during the first study period. Subsequently, status (starting with or without compression) of the participants was listed by the primary investigator (MH) who enrolled participants and instructed them in detail about their status including corresponding dos and don'ts.

#### 2.4.5. Blinding

While participants and investigators are aware of the actual status (with or without compression), research assistants were kept blind to this allocation of the participants and were not allowed to ask, either.

#### 2.4.6. Statistical Analysis

All the eligible participants who were randomly allocated to the two conditions were included in the analysis independently of compliance with the protocol. In order to consider condition (i.e., with and without compression), period (i.e., posttest assessments, 24, 48, 72, and 96 h) and phase effects (i.e., intervention 1 and 2), and primary and secondary endpoints were analyzed using a linear mixed effect model with spherical symmetric within-group correlation also allowing for heteroscedasticity between time-points. The full model included treatment, timepoint, and period as well as their respective two and three-way interactions as fixed effects and subject as random effect. We focused on the adjusted overall (i.e., cumulated effect of all post-intervention assessments) mean difference between the two conditions as the main outcome parameter. Due to the enormous variation for CK and myoglobin, we logarithmized the data of these parameters for further analysis. Significance was accepted at p <0.05.

## 3. Results

**Figure 2:**
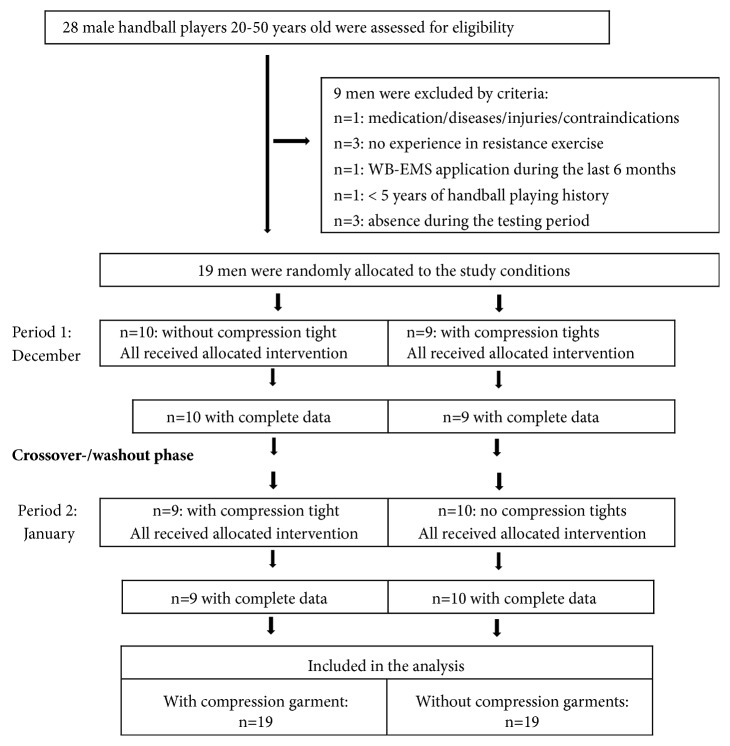
Flow-chart of the study.

**Figure 3:**
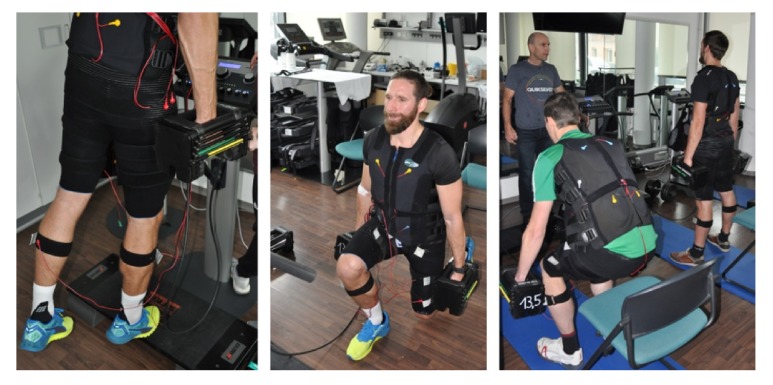
Lower extremity exercises with adjuvant WB-EMS application. Participant shown in Figure 3 gave written informed consent to the presentation of its pictures.

Figure 2 shows participant flow through the study. None of the participants quit the study or was unable to attend a testing session. Further, all 19 participants underwent their allocated treatment and passed through the project strictly according to the study protocol.  Table 1 gives baseline characteristics of the participants.

Participant test compliance averaged RPE 8.3±0.6 (December) and RPE 8.5±0.7 (January) (“7”=really hard, “9”=really, really hard) on the Borg CR-10 scale; furthermore, all the participants completed the prescribed exercise sessions.

Table 2 gives the effect of compression and time after exercise intervention for the primary study endpoint “changes of maximum isokinetic hip- and leg-extensor strength” as determined by an isokinetic leg-press. Of importance, we did not observe any carry-over or phase effects (i.e., December vs. January) for performance and PEPS parameters; thus we did not adjust for this parameter. In summary, based on an intercept of 3955 N, the overall effect of compression tights on exercise-induced changes of maximum isokinetic hip and leg extensor strength was significantly positive (+155 N; p=0.003) (Table 2).

With respect to the development of recovery, pre-exercise maximum strength values were still not reached after 96 h of recovery (-180 N; Table 2; lower section). However, the reductions under compression conditions were significantly less pronounced 24, 48, and 72 h postexercise.

Thus, we confirmed our primary hypothesis that wearing compression tights significantly improves maximum dynamic hip/leg extensor strength after very intense muscular loading.

In parallel to the results on leg extension strength, based on an intercept of 35.6 cm exercise-induced reductions of lower extremity power as determined by a counter movement jump (CMJ) using a force plate were less pronounced (1.21 cm; p<0.001) wearing compression tights (Table 3). Again full recovery for lower extremity muscle power was still not reached 96 h (-2.10 cm; Table 3 lower section) postexercise; however reductions were significantly less pronounced under compression condition 24, 48, 72, and 96 h postexercise.

In general, “Perceived Physical State” (PEPS) was positively affected by the compression garment. However, results vary between the four dimensions of the PEPS. While significant results for “training level” (German version: “Trainiertheit”) (p=0.017) and “flexibility” (p=0.024) were observed, the positive effect of compression did not reach significance (*α*=0.05) for “activation” (German version: “Aktiviertheit”) (p=0.239) and health (p=0.622). All four dimensions of the PEPS converged to baseline levels after 96 h postexercise (p≥0.101). Additionally pain sensation and ailments significantly differ (p=0.002 and p=0.004, respectively) between the conditions with significantly more favourable data for the compression condition. In contrast to the “PEPS” both parameters failed to reach baseline levels 96 h (p=.0.002 and p=0.004) postexercise, independent of the condition.

Table 4 gives raw values for CK. Serum parameters related to EIMD (i.e., CK and myoglobin) significantly increased (p<0.001) during both periods with peak values after 72 h (myoglobin) and 72-96 h (CK), respectively. Peak-CK indicated a severe rhabdomyolysis (i.e., >50-fold increase of baseline values) independently of the condition (Table 4). However, differences were confounded by a repeated bout effect with CK-values about twice higher during the first compared to the second test period. Therefore, we also adjusted for carry over/phase effects and logarithmized the data for the analysis. Applying this complicated procedure, in summary, we observed a significant positive effect of compression on CK (p<0.001), however not on myoglobin (p=0.669).

## 4. Discussion

This randomized controlled crossover study clearly determined the positive effects of compression tights on maximum lower extremity muscle strength and power. Less clear, only two (Training level, Flexibility) out of four PEPS dimensions [23] were significantly impacted by wearing compression tights. Also noteworthy, the positive effect of compression on CK kinetics during recovery was very pronounced, while no significant effect was observed for myoglobin concentration. In summary, however one may argue that the effect of compression tights on the recovery of MIES and lower extremity power is moderate at best. Nevertheless, considering the low effort and time efficiency of this approach along with the aspect that even small differences in performance might decide a narrow match, we think wearing compression tights is a feasible method to accelerate recovery in these athletes.

A considerable number of studies (review in [1–4]) focus on the effect of compression garment on performance parameters during recovery from exercise-induced muscle soreness/DOMS and EIMD. However, due to differences in garment type (i.e., knee socks, calf/thigh/arm sleeves, shorts tights, and leggings), compression parameters, exercise intervention, follow-up periods, and outcomes, definitive evidence of a significantly favorable effect of compression garments on recovery from exercise ((or) muscle soreness/DOMS (and/or) EIMD) has yet to be provided. A recent meta-analysis [3], however, concluded a positive effect of compression garments on “recovery” particularly after resistance exercise and prior strength performance, while the effect of endurance exercise protocols remained non-significant. Indeed, intense eccentric resistance exercise [25] and, to a much higher degree, (very) intense WB-EMS (WB-EMS stimulates all the main muscle group simultaneously with dedicated intensity per muscle group. Considering that muscles can also be stimulated with supramaximal intensity, WB-EMS is ideally suitable [25, 26] for generating very high concentrations of serum parameters related to EIMD.) [17] are known to induce severe muscle soreness/DOMS and/or EIMD particularly in novices. Further, in order to determine the effect of compression on recovery, it is crucial to induce relevant muscle soreness or even EIMD (However, DOMS does not reflect the magnitude of eccentric exercise-induced muscle damage [27]) with corresponding impact on muscular performance. Correspondingly, eccentric resistance training combined with WB-EMS might be an adequate stimulus particularly when unusual for the applicant. Apart from differences in compression garment, recovery parameters and study endpoints, we think the ability to induce relevant muscle soreness, EIMD and sustained reductions of muscle performance might be the reason for the heterogeneous study results in this field [1–4]. Applying conventional endurance exercise intervention with endurance athletes (e.g., [28, 29]) might be a too weak stimulus for generating significant exercise-induced changes of the outcomes addressed. Correspondingly, the power of the predominately low to medium sample size studies in this domain might be too low to determine the slight effects of compression (compared to control) on recovery parameters. One may argue that the quantitative reviews and meta-analyses recently published might overcome this issue [2–4]. However, although important for providing proof of principle of the favorable effect of compression on recovery from exercise, due to the complex interaction of cohort, exercise, compression, recovery, and outcome parameters, only limited practical recommendations for athletes can be drawn from a meta-analysis (see also Gentil et al. [30] who addressed this issue for resistance exercise protocols.).

Due to the above-mentioned complexity, the proper comparison of our results with results of the literature is a daunting task. However, simplifying our approach, a comparison with resistance (or power) exercise trials [9, 11, 12, 31–37] that focus on maximum voluntary contraction (MVC) and/or jump performance as a study outcome might be productive. In summary, conflicting results were reported, with significant faster recovery from resistance exercise [11, 12, 34–36], or failure to observe relevant effects of compression garment [9, 32–34]. Even studies with comparable exercise protocols (i.e., 50-100 maximum eccentric elbow flexions), compression garment, and duration of monitoring (72-96 h post) did not report consistent results [9, 12, 31, 35, 36] (The three latter authors observed significant positive effects, while the first two authors did not report differences between compression and control group). Of importance, results were independent of compression provided by the sleeves ([12, 31] or duration of wearing compression postexercise [9, 31].

Most similar to our study, Goto et al. [34], who applied intense resistance exercise (inter alia 5 sets of 10 reps with 70% 1 RM with 90s of recovery for bilateral leg-press and knee extension, 3 sets 10 reps with 70% 1 RM for calf raises without adjuvant compression; compression not given) with men experienced in resistance training and using whole-body-compression garments worn 24 h immediately postexercise reported significant effects (compression vs. control) on maximum knee extensor strength for the final 24 h postexercise assessment. In parallel, Jakeman et al. [11], who applied intense jumping exercises (1x 10 reps of plyometric drop jumps (60 cm box) with 10 s between jumps and 1 min between sets without adjuvant compression; 17mmHG at the calf, 15 mmHG at the thigh) with moderately exercising women using compression tights worn for 12 h immediately post exercise, determined significant positive effects of compression on leg extension strength and jump performance. In detail, the authors reported significant group differences in favor of compression for squat jump height and maximum isokinetic leg extensor strength following all assessments (24, 48, 72, 96 h post) while the effect on CMJ height was less pronounced and significant at 48 h post-exercise only. Shimokochi et al. [37] also observed significant effects of compression tights (≈20 mmHG at the calf and 10 mmHG at the hip. Data is given by the manufacturer, not validated by the researcher. Compression tights were worn 8 h during overnight sleep) on maximum isokinetic leg extension strength 24 h post exercise (10x 10 reps of maximal isokinetic knee extension contractions, 30 sec of rest). However, the authors did not observe differences in electromyogram (EMG) related variables between the two conditions, thus they concluded neurological factors did not relevantly contribute to the favorable effect of compression tights worn during night sleep on recovery from quadriceps muscle fatigue. In contrast to these positive results, French et al. [33] did not observe significant results on muscular performance (CMJ) 24 or 48 h after an exhausting squat exercise protocol (6x 10 reps with a focus on eccentric movement). Compression tights (12 mmHG at the calf, 10 mmHG at the thigh) were worn 12 h overnight.

Despite being only secondary study endpoints, CK and myoglobin responses warrant brief consideration. Predominately, we determined both parameters to appraise the exercise-induced mechanical and metabolic stress of our protocol and, to a lesser extent, to determine metabolic changes during recovery from exercise. Based on our experience with WB-EMS novices [17] and considering the enhanced training status of the participants (Compared to untrained persons, CK-increase of athletes were much lower [26, 38]), we expected CK and myoglobin concentrations in the area of a moderate (i.e., 10-50-fold increase of resting concentration) rhabdomyolysis [39] after our exercise/lower body EMS protocol that focused exclusively on gluteus, thigh, and calf muscles. However, peak CK and myoglobin concentration in this consistently and closely medically supervised study averaged 34828±24807 IU/l for CK und 3163±2105 *μ*g/l for myoglobin (....however, without any other symptoms as cola colored urine or abnormalities for electrolyte balance) and did thus not relevantly differ from whole body application [17]. Although, the delayed CK and myoglobin peaking after 96 (however, due the termination of FU-assessment 96 h postexercise we are unable to decide whether CK-concentration peaked after 96 h or had still increased….) and 72 h respectively might be a specific feature of WB-EMS application [40] and/or eccentric resistance exercise (e.g., [41]) the need for observational periods longer than 24 or 48 h was supported by this result. Summarizing the effect of compression tights on recovery from mechanical and metabolic stress, our results of positive effects on CK but absence of effects on myoglobin concentration is noteworthy. We are unable to provide a robust explanation of this scenario. We speculate that differences might be due to myoglobin kinetics, specifically the shorter half-life (2–3 hours) and more rapid renal clearance [42]. Further, apart from its (controversial discussed) role as a marker of muscle damage [26], CK plays a vital role in energy control processes [26]. However, considering the long recovery period of 96 h without adjuvant exercise, we do not think that this aspect might be relevant for our results.

Some features and study limitations might decrease the evidence of this trial or at least aggravate its proper interpretation. (1) One main limitation of the project might be that we were unable to validate the correct fit and individual compression gradients using the recognized Kikuhime pressure monitoring device (MediGroup, Melbourne, Australia). However, this was not an aspect of the general validity and reliability of this device [43], but to the proper positioning at the six ankle calf and thigh landmarks proposed. Positioning the device with tape at the anatomical landmark (see [44]) before working the tights higher towards the hip resulted regularly in a displacement of the device. However, this failure to check the proper size of the compression garment based on circumference measurements and correspondingly suggestions of the manufacturer might provide a “real world scenario” for the application of compression tights. (2) The age range of our participants (20-49 years) is quite wide. However, at least when applying mixed RT/WB-EMS protocols we did not observe age- or gender-dependent differences in post exercise pain sensation, ailments, or CK levels [17]. (3) Another limitation might be the rather short washout period of 6 weeks. Indeed, we observed a significant phase effect for serum parameters (CK, myoglobin) with higher concentration during phase 1. Although we are aware of the pronounced repeated bout effect [25] of WB-EMS on CK [17], we (a) still underestimated this effect and (b) had to consider the short, only 8-week transitional phase during the two handball seasons to conduct the project. (4) One may argue that our project was not structured straightforwardly enough. Indeed, the somewhat artificial exercise intervention thwarted the intention to provide a practical approach. Without doubt, although eccentric exercise is a frequent component of handball training, adjuvant WB-EMS application in handball players is still an experimental approach [45]. Applying a more sport-specific intervention (i.e., close to the usual strain type and composition), however, might fail to generate EIMD in these athletes. In contrast, applying intense WB-EMS in “EMS novices” reliably ensures severe muscle soreness and EIMD independently of the training status of the applicants [17]. As discussed above, the need for relevant intervention effects on muscle is crucial for the subsequent effect on recovery, thus we opted to apply an intervention that increases the stimulus of voluntary sport specific movements by electrostimulation. (5) We prescribed an intermitted protocol with 24 h compression immediately postexercise, FU assessments, and subsequently 12 h without compression followed by wearing compression tights overnight for 12 h upwards. This differs from most studies which either applied continuous compression over the whole study period of 24-72 h [31, 32, 34–36] or limited wearing compression garment to 8-12 h (e.g., [9, 11, 37]) or 24h ([12]) of their 24 [37] to -96 h [9, 11, 12] observational period (Unfortunately, not all the authors reported whether FU performance tests were conducted with or without compression garments). (6) The linear mixed effect model applied in this study generated more statistical power to determine group differences compared with simple group comparisons for the particular follow-up tests (i.e., 24, 48, 72, and 96 h postexercise). Unfortunately, this very elaborate and statistically sophisticated procedure prevented a clearly arranged and easy comprehensible presentation of mean values and corresponding dispersion data for the primary and secondary endpoints.

Although the present study confirms the favorable effect of compression garment on recovery from exercise [3], the exact mechanism behind recovery from EIMD is still unclear. Undoubtedly, several hemodynamic and cellular areas with an impact on EIMD are affected by external compression [3]. This includes reductions in cellular trauma alongside swelling, muscle vibration and perceived muscle pain, enhanced circulation, blood lactate removal, and increases in body temperature [3, 46]. However, the relevance of these factors may vary between specific forms of exercise [3]; thus it is essential that further studies investigate the mechanisms of compression-induced effects on recovery from EIMD.

## 5. Conclusion

In summary, we observed a significant positive effect of compressive tights on performance parameters after resistance-type exercise induced muscle damage (EIMD). Based on our results we recommend athletes to wear compression tights immediately 24 h postexercise and further intermittently with an overnight break during the day, particularly after intense exercise with a pronounced eccentric aspect.

## Figures and Tables

**Table 1:** Baseline characteristics of the cohort.

**Variable**	**Participants (n=19)**	**min - max**
	**MV ± SD**	
Age [years]	31.3 ± 7.7	20 - 49

Body Height [cm]	183.5 ± 8.6	170 - 202

Body weight [kg]	81.1 ± 7.6	67.6 – 96.8

Lean Body Mass [kg]	69.3 ± 8.5	56.2 - 87.5

Percent Body Fat [%]	14.6 ± 5.6	7.7 – 26.3

Physical activity [Index]	4.26 ± 1.56	1 - 6

Training volume [min/week]	176 ± 64	120 - 300

Smoker [n]	0	-* *-* *-* *-* *-* *-* *-* *-* *-* *-

**Table 2:** Intercept and fixed effect (bold text) results for maximum hip/leg extension strength (in [N]). Lower part of the table: overall reduction of maximum hip/leg extension strength (in [N]) during the postexercise period of 96 h. MV: mean value, SE: standard error; DF: degrees of freedom; 95-CI: 95% confidence interval.

Variable [N]	MV	SE	DF	95%-CI	p
Intercept	3955	179	157	3601 to 4309	-* *-* *-* *-* *-

**Compression**	**155**	**51**	**157**	**54 to 256**	**0.003**

24 h post	-358	72	157	-216 to -499	<0.001

48 h post	-303	78	157	-149 to -457	<0.001

72 h post	-228	78	157	-73 to 383	0.004

96 h post	-180	75	157	-32 to -327	0.017

**Table 3:** Intercept and fixed effect (bold text) results for lower extremity power (in [cm]). Lower part of the table: overall reduction lower extremity power (in [cm]) during the postexercise period of 96 h. MV: mean value; SE: standard error; DF: degrees of freedom; 95-CI: 95% confidence interval.

Variable [cm]	MV	SE	DF	95%-CI	p
Intercept	35.6	1.3	157	32.9 to 38.2	-* *-* *-* *-* *-

**Compression**	**1.21**	**0.31**	**157**	**0.60 to 1.82**	**<0.001**

24 h post	-2.59	0.48	157	-1.64 to -3.53	<0.001

48 h post	-3.32	0.57	157	-2.20 to -4.43	<0.001

72 h post	-2.87	0.60	157	-1.70 to -4.05	<0.001

96 h post	-2.10	0.50	157	-1.12 to -3.09	<0.001

**Table 4:** Raw data for creatine kinase (IU/l) at baseline and 24 h, 48 h, 72 h and 96 h postexercise.

compression	baseline	24 h	48 h	72 h	96 h	Peak
	MV ± SD;	MV ± SD;	MV ± SD;	MV ± SD;	MV ± SD;	MV ± SD
**Creatine kinase (CK) [IU/l]**						

Without	228 ± 86	4910±6941	12735±14637	29878±24096	31755±24136	34828±24807

With	214 ± 82	2310±3599	7018±7531	15453±12940	14700±9722	17593±13086

## Data Availability

The data used to support the findings of this study are available from the corresponding author upon request.

## References

[1] MacRae B. A., Cotter J. D., Laing R. M. (2011). Compression garments and exercise: garment considerations, physiology and performance. *Sports Medicine*.

[2] Engel F. A., Holmberg H.-C., Sperlich B. (2016). Is There Evidence that Runners can Benefit from Wearing Compression Clothing?. *Sports Medicine*.

[3] Brown F., Gissane C., Howatson G., van Someren K., Pedlar C., Hill J. (2017). Compression Garments and Recovery from Exercise: A Meta-Analysis. *Sports Medicine*.

[4] Hill J., Howatson G., van Someren K., Leeder J., Pedlar C. (2014). Compression garments and recovery from exercise-induced muscle damage: a meta-analysis. *British Journal of Sports Medicine*.

[5] Marqués-Jiménez D., Calleja-González J., Arratibel I., Delextrat A., Terrados N. (2016). Are compression garments effective for the recovery of exercise-induced muscle damage? A systematic review with meta-analysis. *Physiology & Behavior*.

[6] Hill K. D., Hunter S. W., Batchelor F. A., Cavalheri V., Burton E. (2015). Individualized home-based exercise programs for older people to reduce falls and improve physical performance: A systematic review and meta-analysis. *Maturitas*.

[7] Argus C. K., Driller M. W., Ebert T. R., Martin D. T., Halson S. L. (2013). The effects of 4 different recovery strategies on repeat sprint-cycling performance. *International Journal of Sports Physiology and Performance*.

[8] Chatard J.-C., Atlaoui D., Farjanel J., Louisy F., Rastel D., Guézennec C.-Y. (2004). Elastic stockings, performance and leg pain recovery in 63-year-old sportsmen. *European Journal of Applied Physiology*.

[9] Cerqueira M. S., Borgesa L. S., dos Santos Rocha J. A. (2014). Twelve hours of a compression sleeve is not enough to improve the muscle recovery of anexercise-damaged upper arm. *Apunts Medicina de l'Esport*.

[10] Gill N. D., Beaven C. M., Cook C. (2006). Effectiveness of post-match recovery strategies in rugby players. *British Journal of Sports Medicine*.

[11] Jakeman J. R., Byrne C., Eston R. G. (2010). Lower limb compression garment improves recovery from exercise-induced muscle damage in young, active females. *European Journal of Applied Physiology*.

[12] Kim J., Kim J., Lee J. (2017). Effect of compression garments on delayed-onset muscle soreness and blood inflammatory markers after eccentric exercise: A randomized controlled trial. *Journal of Exercise Rehabilitation*.

[13] Perrey S., Bringard A., Racinais S., Puchaux K., Belluye N. (2008). Graduated Compression Stockings and Delayed Onset Muscle Soreness. *The Engineering of Sport*.

[14] Webb E. C., Willems M. E. (2010). Effects of Wearing Graduated Compression Garment during Eccentric Exercise. *Medicina Sportiva*.

[15] World Medical Association (2013). World Medical Association declaration of Helsinki ethical principles for medical research involving human subjects. *Journal of the American Medical Association*.

[16] Moher D., Hopewell S., Schulz K. F. (2010). CONSORT 2010 explanation and elaboration: updated guidelines for reporting parallel group randomised trials.. *BMJ*.

[17] Teschler M., Weissenfels A., Frohlich M. (2016). Very high creatine kinase CK levels after WB_EMS. Are there implications for health. *International Journal of Clinical and Experimental Medicine*.

[18] Steele J., Fisher J., Giessing J., Gentil P. (2017). Clarity in reporting terminology and definitions of set endpoints in resistance training. *Muscle & Nerve*.

[19] Kemmler W., Weissenfels A., Willert S. (2018). Efficacy and Safety of Low Frequency Whole-Body Electromyostimulation (WB-EMS) to Improve Health-Related Outcomes in Non-athletic Adults. A Systematic Review. *Frontiers in Physiology*.

[20] Borg E., Kaijser L. (2006). A comparison between three rating scales for perceived exertion and two different work tests. *Scandinavian Journal of Medicine & Science in Sports*.

[21] Kemmler W., Roloff I., Baumann H. (2006). Effect of exercise, body composition, and nutritional intake on bone parameters in male elite rock climbers. *International Journal of Sports Medicine*.

[22] Kemmler W., Bebenek M., Stengel S., Kohl M., Bauer J. (2015). Increases of Cardiometabolic Risk in Young Adults. Impact of Exercise Reductions during the College Years. *British Journal of medicine and Medical Research*.

[23] Kleinert J. (2006). Adjektivliste zur Erfassung der Wahrgenommenen Körperlichen Verfassung (WKV). *Zeitschrift für Sportpsychologie*.

[24] Wittke A., Von Stengel S., Hettchen M. (2017). Protein supplementation to augment the effects of high intensity resistance training in untrained middle-aged males: The randomized controlled PUSH trial. *BioMed Research International*.

[25] Koch A. J., Pereira R., Machado M. (2014). The creatine kinase response to resistance exercise. *Journal of Musculoskeletal and Neuronal Interactions*.

[26] Baird M. F., Graham S. M., Baker J. S., Bickerstaff G. F. (2012). Creatine-Kinase- and Exercise-Related Muscle Damage Implications for Muscle Performance and Recovery. *Journal of Nutrition and Metabolism*.

[27] Nosaka K., Newton M., Sacco P. (2002). Delayed-onset muscle soreness does not reflect the magnitude of eccentric exercise-induced muscle damage. *Scandinavian Journal of Medicine & Science in Sports*.

[28] Ali A., Caine M. P., Snow B. G. (2007). Graduated compression stockings: Physiological and perceptual responses during and after exercise. *Journal of Sports Sciences*.

[29] Bieuzen F., Brisswalter J., Easthope C., Vercruyssen F., Bernard T., Hausswirth C. (2014). Effect of wearing compression stockings on recovery after mild exercise-induced muscle damage. *International Journal of Sports Physiology and Performance*.

[30] Gentil P., Arruda A., Souza D. (2017). Is there any practical application of meta-analytical results in strength training?. *Frontiers in Physiology*.

[31] Carling J. C., Francis K., Lorish C. (1995). The effects of continuous external compression on delayed-onset muscle soreness (DOMS). *International Journal of Rehabilitation and Health*.

[32] Davies V., Thompson K. G., Cooper S. M. (2009). The effects of compression garments on recovery. *The Journal of Strength and Conditioning Research*.

[33] French D. N., Thompson K. G., Garland S. W. (2008). The effects of contrast bathing and compression therapy on muscular performance. *Medicine & Science in Sports & Exercise*.

[34] Goto K., Morishima T. (2014). Compression garment promotes muscular strength recovery after resistance exercise. *Medicine & Science in Sports & Exercise*.

[35] Kraemer W. J., Bush J. A., Wickham R. B. (2001). Influence of compression therapy on symptoms following soft tissue injury from maximal eccentric exercise. *Journal of Orthopaedic & Sports Physical Therapy*.

[36] Kraemer W. J., Bush J. A., Wickham R. B. (2001). Continuous compression as an effective therapeutic intervention in treating eccentric-exercise-induced muscle soreness. *Journal of Sport Rehabilitation *.

[37] Shimokochi Y., Kuwano S., Yamaguchi T., Abutani H., Shima N. (2017). Effects of Wearing a Compression Garment During Night Sleep on Recovery From High-Intensity Eccentric-Concentric Quadriceps Muscle Fatigue. *The Journal of Strength and Conditioning Research*.

[38] Malone S., Mendes B., Hughes B. (2018). Decrements in Neuromuscular Performance and Increases in Creatine Kinase Impact Training Outputs in Elite Soccer Players. *The Journal of Strength and Conditioning Research*.

[39] Visweswaran P., Guntupalli J. (1999). Rhabdomyolysis. *Critical Care Clinics*.

[40] Kemmler W., Teschler M., Bebenek M., von Stengel S. (2015). (Very) high Creatinkinase concentration after exertional whole-body electromyostimulation application: health risks and longitudinal adaptations.. *Wiener Medizinische Wochenschrift*.

[41] Rodenburg J. B., Bar P. R., De Boer R. W. (1993). Relations between muscle soreness and biochemical and functional outcomes of eccentric exercise. *Journal of Applied Physiology*.

[42] Berridge R. B., Bolon B. E., Herman E., Wallig M. A., Bolon B., Haschek W. M., Rousseaux C. G., Mahle B. W. (2018). Skeletal Muscle System. *Fundamentals of Toxicologic Pathology*.

[43] Brophy-Williams N., Driller M. W., Halson S. L., Fell J. W., Shing C. M. (2014). Evaluating the Kikuhime pressure monitor for use with sports compression clothing. *Sports Engineering*.

[44] Brophy-Williams N., Driller M. W., Shing C. M., Fell J. W., Halson S. L. (2015). Confounding compression: the effects of posture, sizing and garment type on measured interface pressure in sports compression clothing. *Journal of Sports Sciences*.

[45] Kemmler W., Teschler M., Bebenek M., von Stengel S. (2015). Hohe Kreatinkinase-Werte nach exzessiver Ganzkörper-Elektromyostimulation: gesundheitliche Relevanz und Entwicklung im Trainingsverlauf. *Wiener Medizinische Wochenschrift*.

[46] Born D.-P., Sperlich B., Holmberg H.-C. (2013). Bringing light into the dark: effects of compression clothing on performance and recovery. *International Journal of Sports Physiology and Performance*.

